# Potential Effect of Intravesical Platelet‐Rich Plasma Injection on Urinary Retention: A Case Report

**DOI:** 10.1002/ccr3.71278

**Published:** 2025-10-21

**Authors:** Kangji Liao, Qiguang Li, Xianlin Yi

**Affiliations:** ^1^ Department of Urology WuMing Hospital of Guangxi Medical University Nanning China; ^2^ Department of Urology Maternal and Child Health Hospital of Hubei Province, Tongji Medical College, Huazhong University of Science and Technology Wuhan China; ^3^ Department of Urology Xianhu Campus of the First Affiliated Hospital of Guangxi University of Traditional Chinese Medicine Nanning China

**Keywords:** acute radiation cystitis, growth factors, platelet‐rich plasma (PRP), urinary retention

## Abstract

Pelvic autonomic nerve injury during radical treatment of cervical cancer and postoperative radiotherapy leads to bladder dysfunction and urinary retention. At present, there is no specific treatment. We report a 51‐year‐old patient with cervical adenocarcinoma who developed acute radiation cystitis and urinary retention 2 months after radiotherapy. We used three courses of intravesical injection of platelet‐rich plasma, which improved the patient's bladder emptying function and restored the bladder mucosa. We believe that intravesical injection of platelet‐rich plasma can not only be used to treat acute radiation cystitis, but also be a potential treatment for urinary retention after pelvic tumor surgery. However, further controlled clinical trials are needed to confirm.

## Graphical abstract

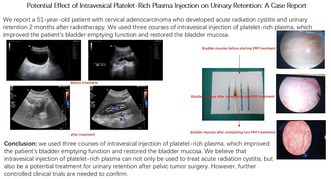

## 1 Introduction

Postoperative radiotherapy is one of the measures to improve the survival rate of patients with cervical cancer, but this regimen can also cause a variety of toxic side effects and complications during treatment. Radiation cystitis is a common complication, with a prevalence of about 5% [1]. Radiation cystitis is classified as acute or chronic, with a cut off of 3 months from the start of radiotherapy. Acute radiation cystitis is characterized by injury to the bladder mucosa, with symptoms of painful urination, urinary urgency, frequency, dysuria, and urinary tract infections also being present. The condition is usually self‐limiting [2]. Chronic radiation cystitis can occur months to years after radiotherapy and lasts for varying periods, and in severe cases, a bladder fistula can develop. Currently, the treatment of acute radiation cystitis is mainly based on the anti‐inflammatory, hemostatic, internal administration of anticholinergic drugs and symptomatic supportive means such as bladder drug perfusion and hyperbaric oxygen therapy.

Platelet‐rich plasma (PRP) is a platelet‐rich blood product extracted by centrifugation from the patient's own blood. The healing properties of PRP have been well documented. Platelets contain three types of granules—alpha, delta, and lysosomes—of which the alpha granules are the most distinctive and abundant type and contain many platelet secretory bodies [3]. After PRP activation, α‐granules begin to release large amounts of growth factors and cytokines such as vascular endothelial growth factor, tumor growth factor β, platelet‐derived growth factor, and endothelial growth factor within 10 min [4]. These factors act synergistically to promote angiogenesis, fibroproliferation, and epithelial reconstruction [5, 6].

Urinary retention is currently a difficult clinical problem that is poorly treated. We describe a clinical case of acute radiation cystitis treated with intravesical platelet‐rich plasma injections in a 51‐year‐old patient with stage IB1 invasive adenocarcinoma of the cervix.

### 1.1 Clinical History

A 51‐year‐old patient was diagnosed with cervical cancer by hysteroscopy + segmental diagnostic scraping, and underwent a hysterectomy of the uterus and adnexa, pelvic lymph node dissection, and para‐abdominal lymph node dissection under general anesthesia because of “feeling of menstrual bleeding for 1 month.” Postoperative diagnosis was invasive adenocarcinoma of the cervix, stage IB1 (pT1b1N0M0 IB1). Postoperative pelvic radiotherapy was performed with a regimen of PTV‐CTV = 5040 cGy/28F for 3 cycles. During this period, III° myelosuppression and radiation enteritis developed, and she was given a white‐liquid boosting and retention enema treatment. Subsequently, the patient began to experience frequent urination, urgency, and pain, as well as nighttime urinary leakage. After oral administration of 5 mg/day of Solifenacin and bladder instillation of 5 mg of dexamethasone and 80 mg of gentamicin, the symptoms did not improve. The patient developed severe lower abdominal distension and pain, and difficulty urinating worsened.

### 1.2 Investigations

**FIGURE 1:**
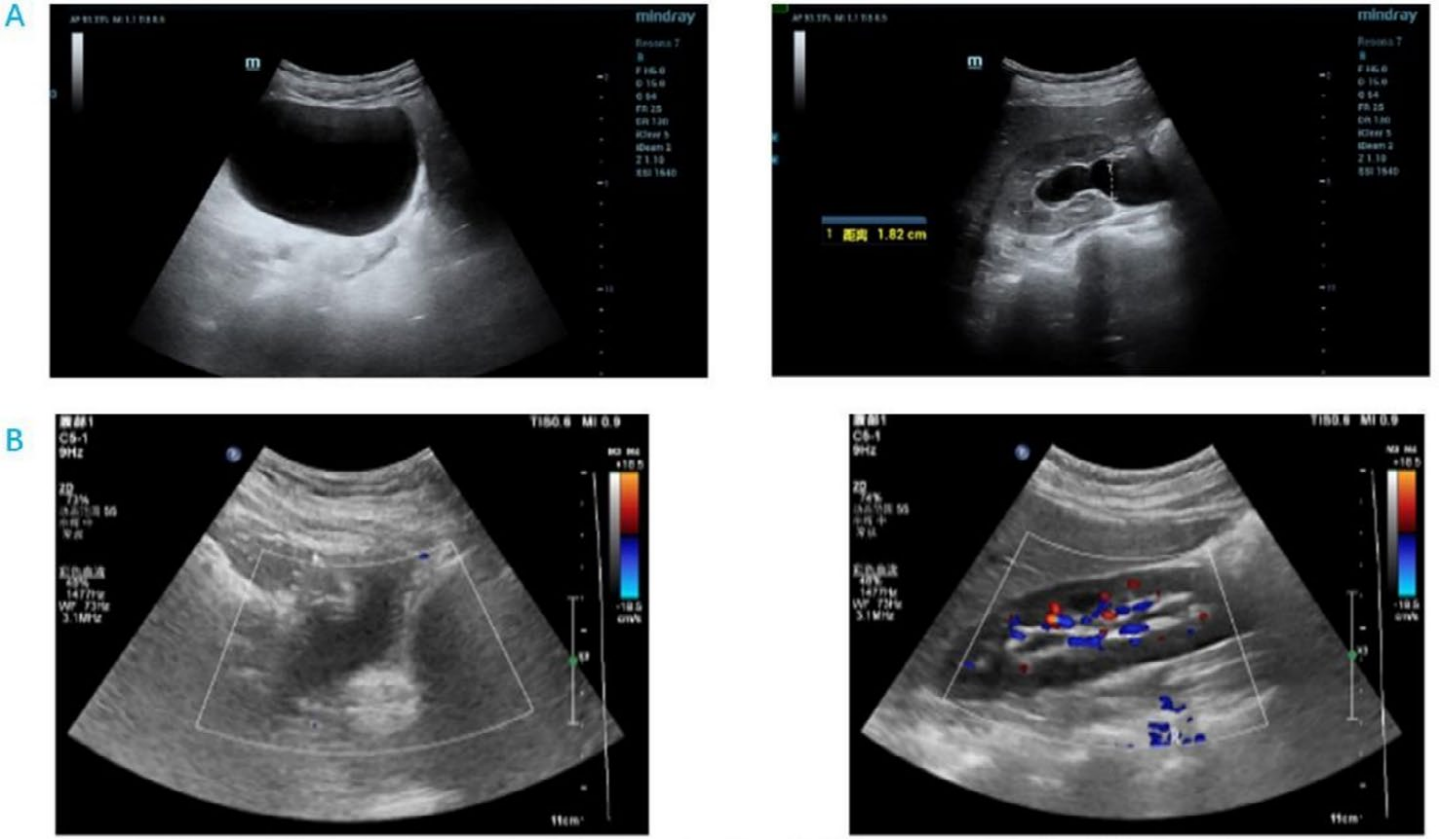
Ultrasound of the urinary tract before starting PRP treatment (A), Ultrasound of the urinary tract after completion of PRP treatment (B).

Laboratory tests, including complete blood cell count: red blood cell, white blood cell, platelet, and lymphocyte counts all decreased, and urine analysis: 46 white blood cells, 2634 bacteria (Table 1). Results of urological ultrasound + residual urine volume measurement: increased residual urine in the separated bladder of both renal collecting systems (residual urine volume 459.8 mL) (Figure 1A). A urinary catheter was inserted and left in place for a prolonged period. The patient was diagnosed with acute radiation cystitis and went on to complete radiotherapy.

**TABLE 1:** Treatment time and partial examination results.

Number of treatments and surgical duration	Abnormal results of urine routine examination	Residual urine and bladder size measured by urinary tract ultrasound
The first time	46 white blood cells	459.8 mL
September 11, 2024	2634 bacteria	10.9 × 11.4 × 7.4 cm
The second time October 17, 2024	—	34 mL 5.0 × 3.9 × 3.5 cm
The third time November 14, 2024	181 white blood cells	12 mL 2.7 × 2.8 × 3.4 cm

### 1.3 Outcome

**FIGURE 2:**
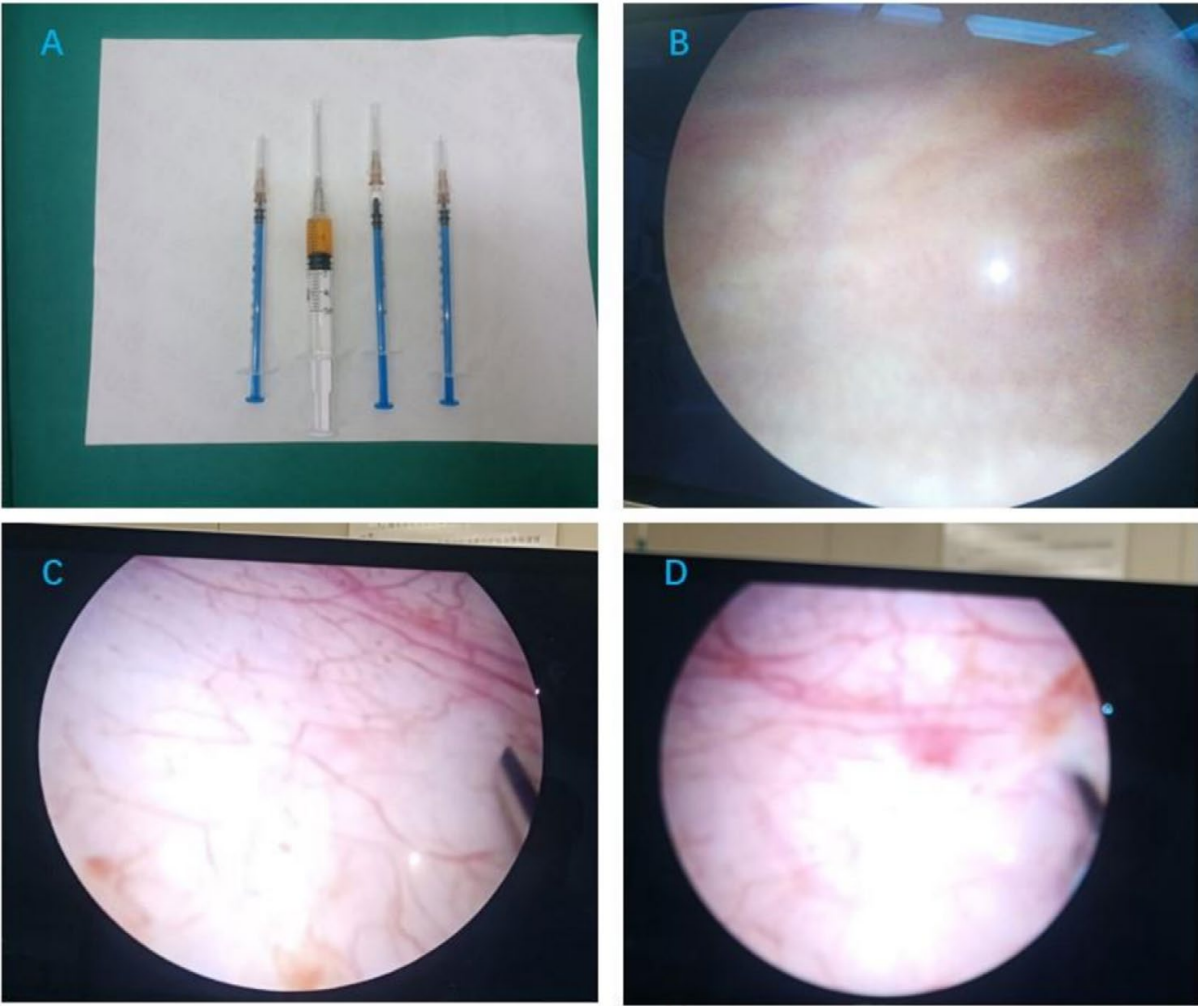
PRP obtained from centrifuged patient blood (A), Bladder mucosa before starting PRP treatment (B), and Bladder mucosa after completing one PRP treatment (C, D).

**FIGURE 3:**
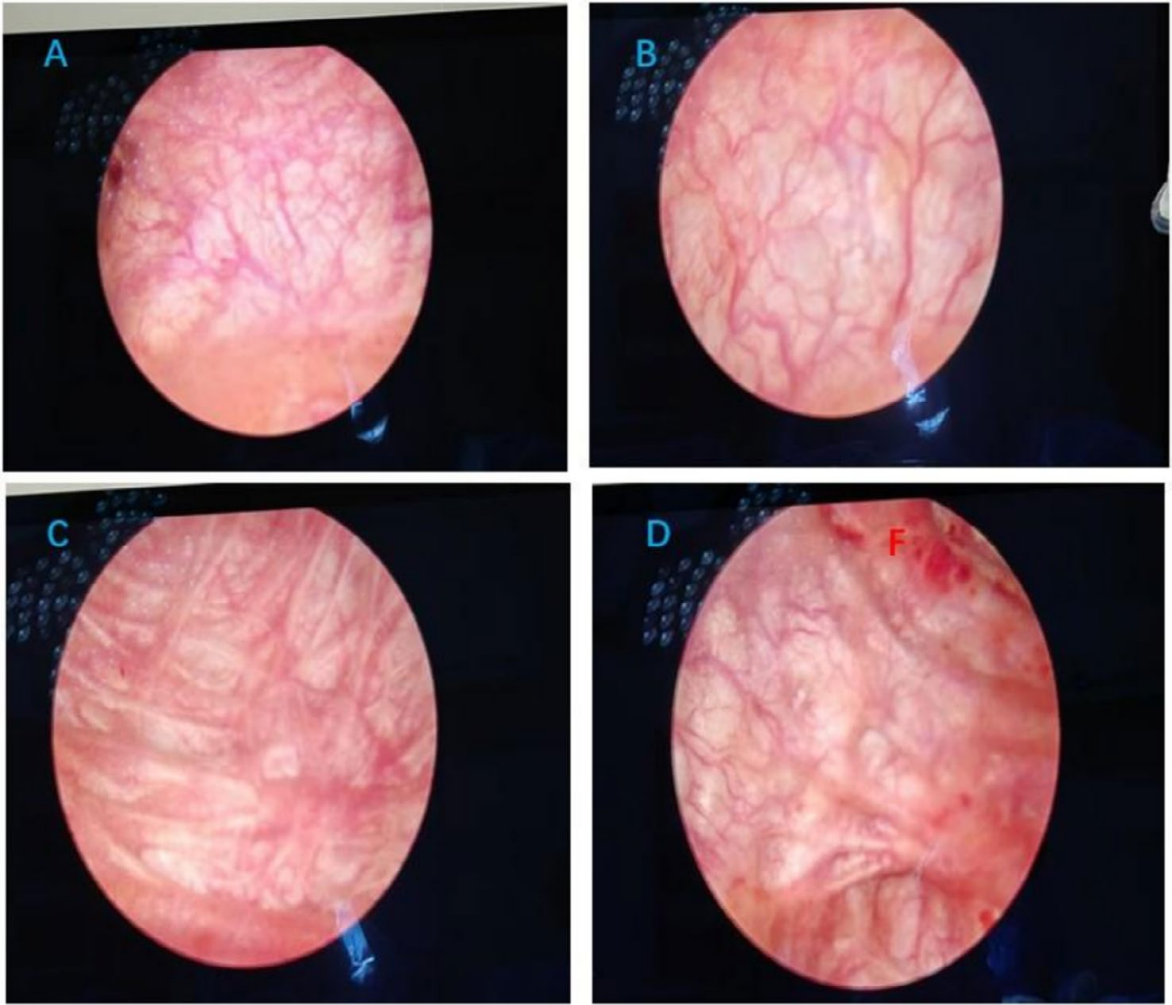
Bladder mucosa after completing two PRP treatments (A–D).

We treated this patient with 3 cycles of intravesical platelet‐rich plasma injections in the lithotomy position, instillation of lidocaine 100 mg and ropivacaine 100 mg for local infiltration anesthesia of the urethra and bladder, and injections of platelet‐rich plasma obtained by centrifugation of the patient's blood at 10 points within the bladder wall under direct cystoscopic vision (Figure 2). The bladder mucosa recovered from its previous pallor with edema and follicles to a smooth mucosa with a rich blood supply (Figure 3). Urological ultrasound + residual urine volume measurement examination showed a reduction in residual urine volume from 459.8 to 34 mL and a residual urine volume of 12 mL after completion of phase 3 treatment (Figure 1B); platelet‐rich plasma injections had a great improvement in bladder emptying, and the patient's voiding function was basically back to normal (Refer to Table 1).

## 2 Discussion

Radiotherapy causes damage to normal cells of the urinary tract of the bladder and fibrosis of blood vessels and tissue cells. The bladder mucosa is damaged by the radioactive material and experiences varying degrees of pain and discomfort, leaving the bladder in a state of inflammatory infiltration such as congestion, necrosis, hemorrhage, edema, and ulceration. Haematuria is a typical symptom of acute radiation cystitis and may manifest as both microscopic and macroscopic haematuria. Throughout the treatment period, the patient's routine urinalysis revealed leukocytes and bacteria and did not reveal any red blood cells.

Removal of the uterus and adnexa, pelvic lymph node dissection, and para‐abdominal aortic lymph node dissection may partially disrupt the nerve and blood supply to the patient's bladder, resulting in a decrease in the elastic muscle fibers of the bladder wall and weakness of the bladder's forcible muscle contractions, leading to neurogenic bladder dysfunction. The smooth muscle of the bladder is also radiosensitive. After radiation therapy, normal smooth muscle may be replaced by fibroblasts, ultimately leading to increased collagen deposition, decreased bladder compliance, and functional changes. The double effects of pelvic surgery injury and radiotherapy further aggravate dysuria, resulting in urinary retention.

Radiation cystitis is often a complication of pelvic radiotherapy, and this process is one of the long‐term chronic recurrence symptoms. Currently, no treatment is very successful [7]. The goals of treatment for acute radiation cystitis are primarily to relieve symptoms, promote inflammatory regression, and minimize the impact on the patient's quality of life. The main focus is on symptomatic support, such as medication, cystoscopic electrocoagulation for hemostasis, hyperbaric oxygen therapy, etc. [8]. However, medication has certain side effects, and hyperbaric oxygen therapy requires certain hardware facilities and is prone to relapse after treatment. Most patients use intermittent catheterization or long‐term indwelling urinary catheters to relieve symptoms of urinary retention, but patients develop bone marrow suppression and low immunity after radiotherapy, and the catheterization approach further increases the risk of urinary tract infections. PRP accelerates tissue repair and regeneration because platelets are enriched with a variety of growth factors, such as platelet‐derived growth factor (PDGF), transforming growth factor‐beta (TGF‐β), and epidermal growth factor (EGF) [9]. PDGF is also important in recruiting pericytes into capillaries, thereby increasing their structural integrity and promoting angiogenesis [10]. TGF‐β can also enhance the chemotaxis of fibroblasts and smooth muscle cells while regulating the expression of collagen and collagenase, which can lead to inflammation, angiogenesis, reepithelialization, connective tissue regeneration, granulation tissue formation, matrix formation, and tissue remodeling. PRP is beneficial for angiogenesis and skin graft growth, inhibits fibrosis formation and inflammation infiltration into the muscle layer, suppresses scar formation in the bladder muscle layer, and partially improves bladder capacity [11]. These growth factors directly promote basal cell proliferation and restore bladder mucosal epithelial barrier function; inhibit the release of inflammatory factors while promoting the repair of bladder nerves [12]; by regulating collagen metabolism, inhibiting scar formation in the bladder muscle layer, and partially improving bladder capacity [13]. In addition, PRP promotes the formation of new blood vessels and improves local blood circulation in the bladder, thus providing a better supply of nutrients and waste removal from damaged tissues. After PRP treatment, the patient's bladder mucosa was significantly improved. Cystoscopy showed that the bladder mucosa was smooth and pink, with a network of tiny blood vessels on the surface, and there was no obvious congestion, oedema, ulceration, or other abnormalities (Figure 2). PRP uses the patient's own blood components, so it can reduce the risk of infection and immune rejection, and avoid the side effects of drug therapy.

After about 3 months of the entire PRP treatment cycle, the patient's symptoms were largely restored, and at the end of the second treatment cycle, the patient's residual urine output was significantly reduced. Although acute radiation cystitis is usually self‐limiting, based on the patient's clinical examination and recovery after treatment, as well as literature reports on the therapeutic effects of PRP in other diseases [13, 14, 15], we believe that PRP is not only a treatment for acute radiation cystitis but also a potential treatment for postoperative urinary retention after pelvic tumor surgery.

The disadvantage of our study is the lack of high‐quality images due to the partial loss of the surgical video and photographs of this patient prior to PRP injection treatment; however, we still have blurry photographs and clear imaging evidence. In addition, it is uncertain whether the improvement in urinary retention is entirely attributable to the patient's own ability to repair the condition; however, the need for an indwelling urinary catheter prior to treatment in this patient suggests that it is not entirely due to the patient's own ability to repair the condition.

## 3 Conclusion

PRP has its unique advantages and wide application prospects in theory, and intravesical platelet‐rich plasma injection can not only treat acute radiation cystitis but also be a potential treatment for postoperative urinary retention after pelvic tumor surgery. However, further controlled clinical trials are needed to confirm the efficacy of intravesical platelet‐rich plasma injection for acute radiation cystitis and to promote its wider clinical application.

## Author Contributions

**Kangji Liao:** conceptualization, data curation, writing – original draft. **Qiguang Li:** conceptualization, methodology. **Xianlin Yi:** funding acquisition, writing – review and editing.

## Consent

Written informed consent was obtained from the patient for publication of this case report and any accompanying images.

## Conflicts of Interest

The authors declare no conflicts of interest.

## Supporting information


**Appendix S1:** Abstract supplementary document.

## Data Availability

The data that support the findings of this study are available from the first author upon reasonable request.
