# Supplementary material for: Potential Effect of Intravesical Platelet‐Rich Plasma Injection on Urinary Retention: A Case Report
Source: Clin Case Rep. 2025 Oct 21;13(10):e71278. doi: 10.1002/ccr3.71278 (PMC12538232; doi:10.1002/ccr3.71278)
Supplement: Supplementary file 1 — Appendix S1: Abstract supplementary document. [file CCR3-13-e71278-s001.docx]

**Abstract:**

Pelvic autonomic nerve injury during radical treatment of cervical cancer and postoperative radiotherapy leads to bladder dysfunction and urinary retention. At present, there is no specific treatment. We report a 51-year-old patient with cervical adenocarcinoma who developed acute radiation cystitis and urinary retention 2 months after radiotherapy. We used three courses of intravesical injection of platelet-rich plasma, which improved the patient's bladder emptying function and restored the bladder mucosa. We believe that intravesical injection of platelet-rich plasma can not only be used to treat acute radiation cystitis, but also be a potential treatment for urinary retention after pelvic tumor surgery. However, further controlled clinical trials are needed to confirm.
